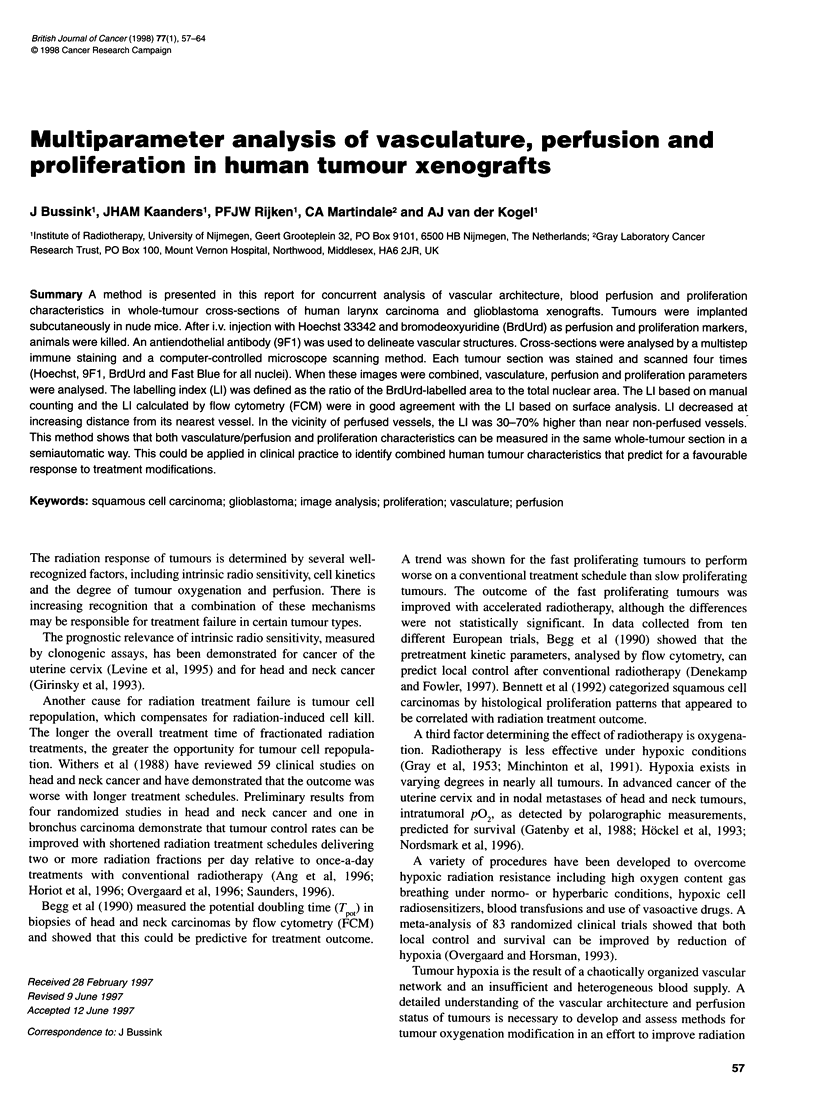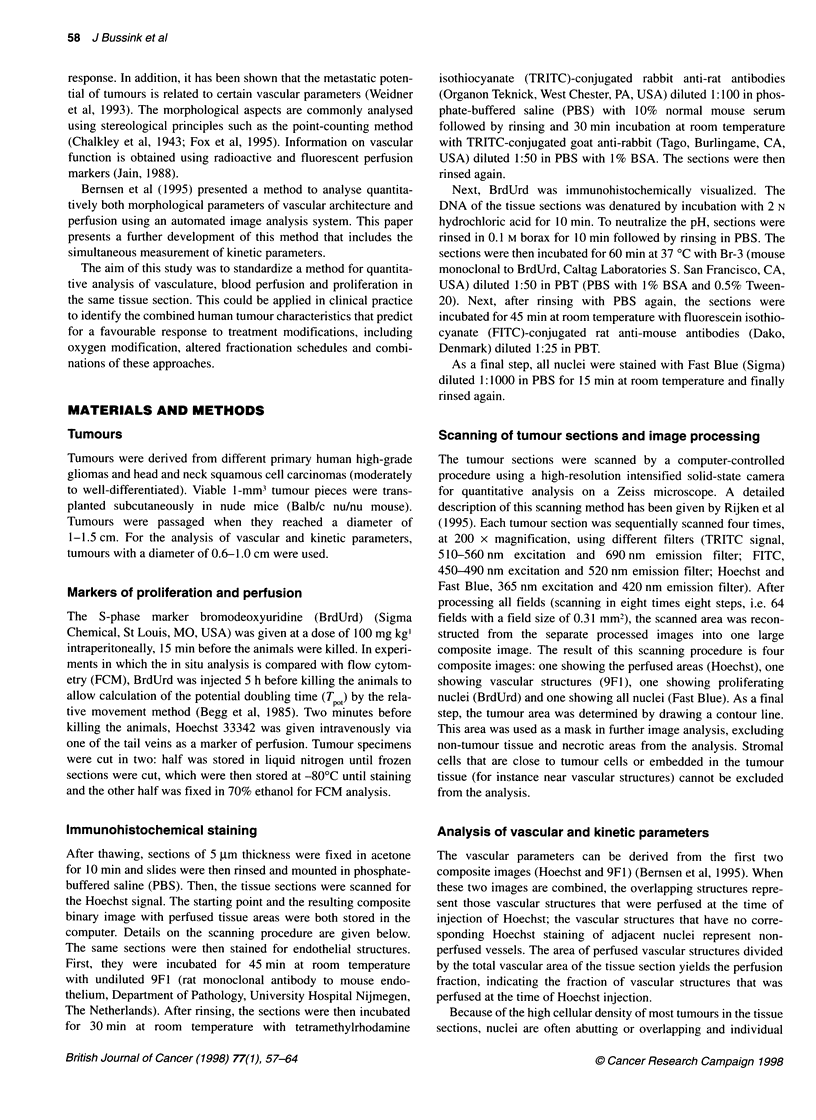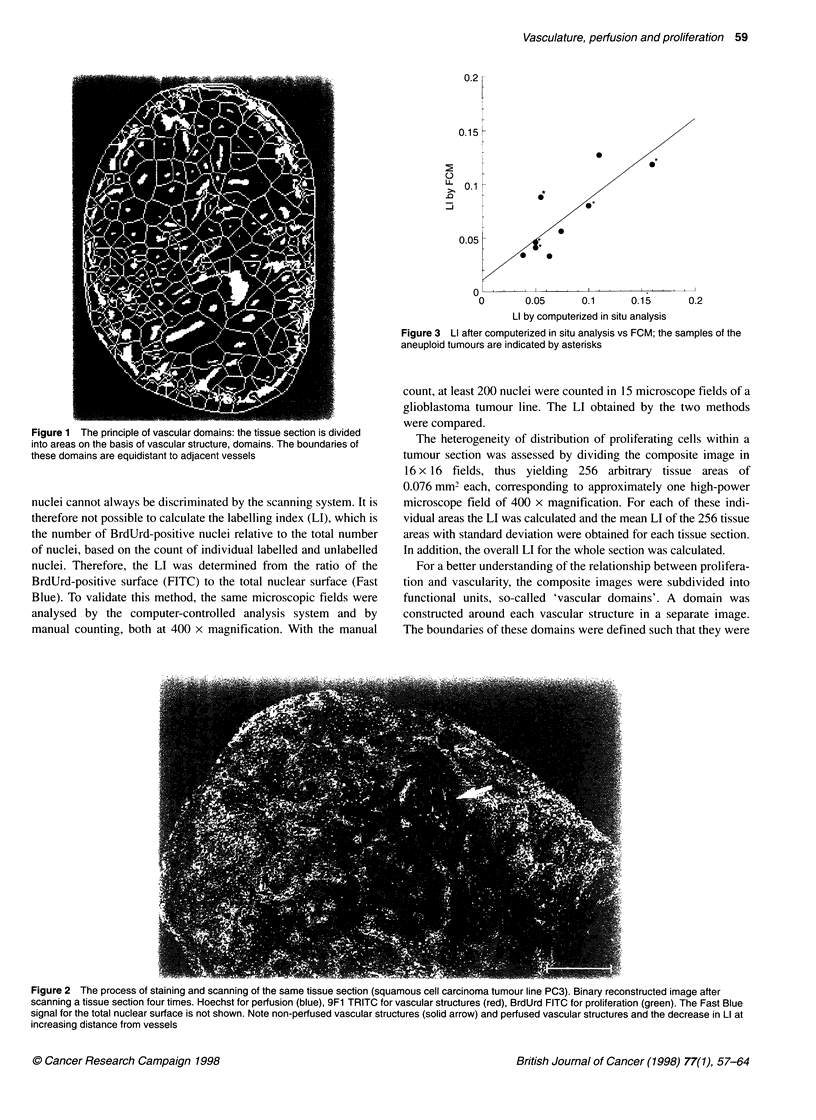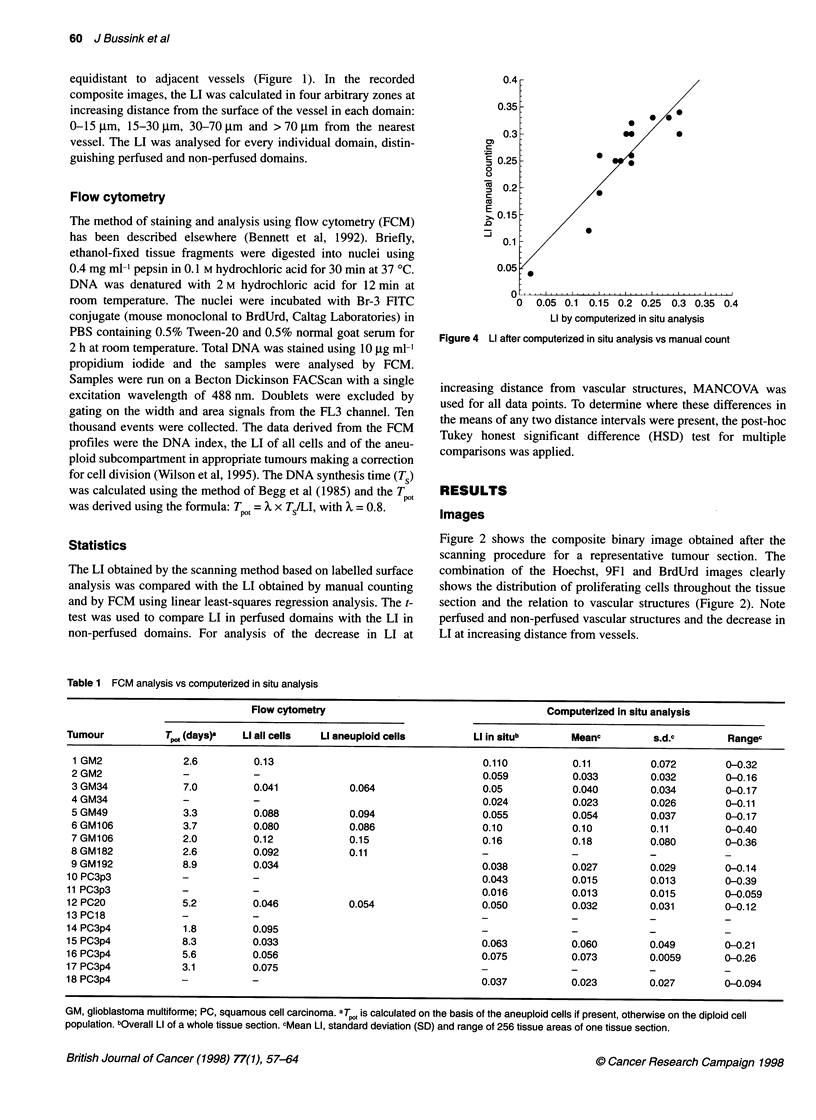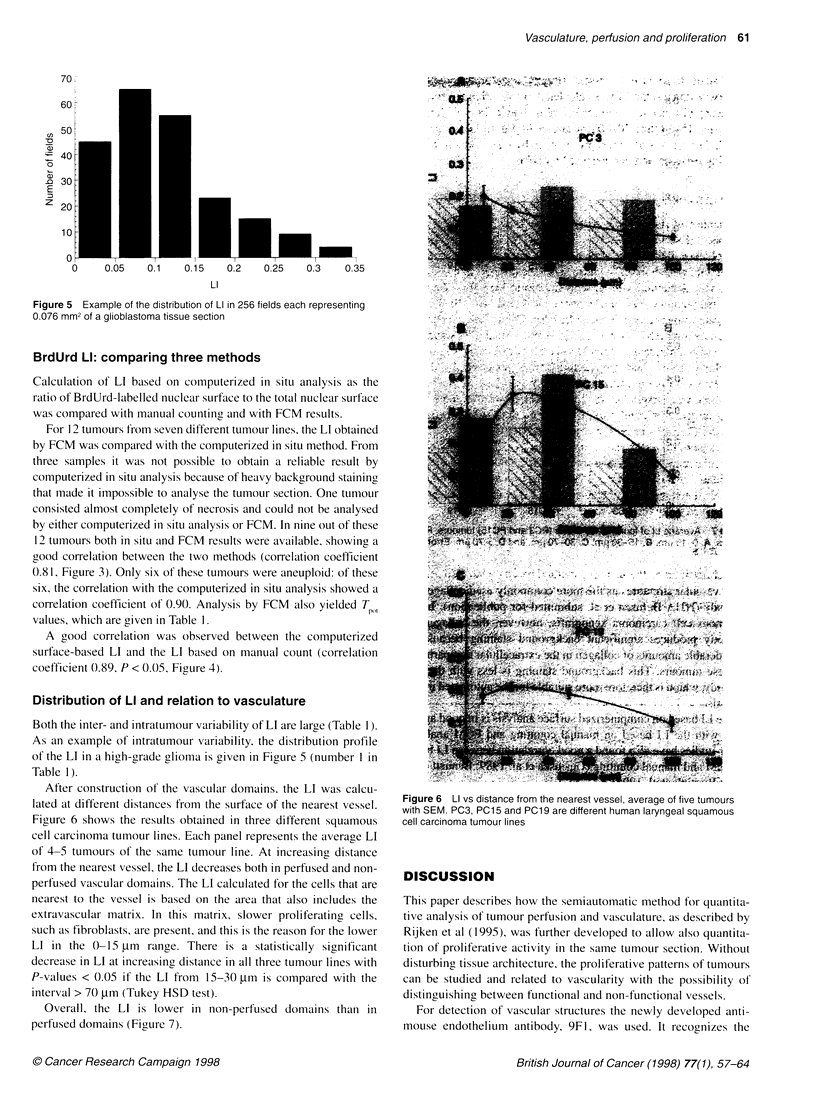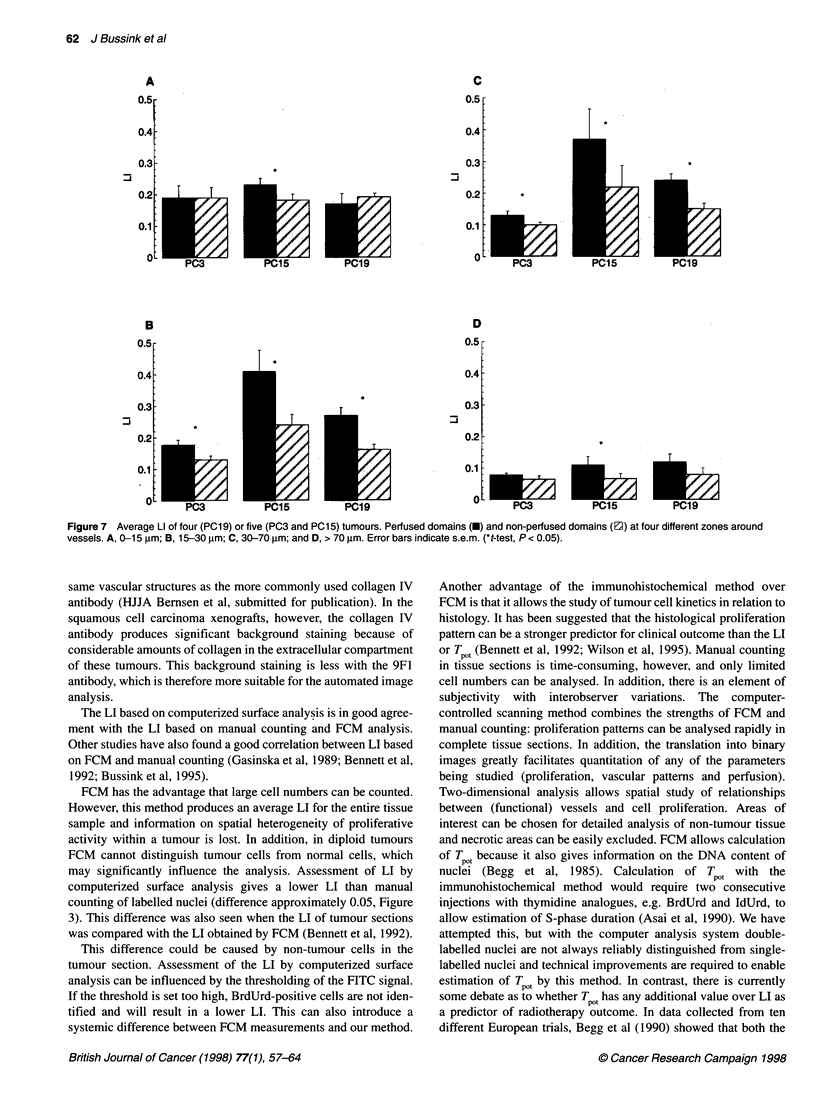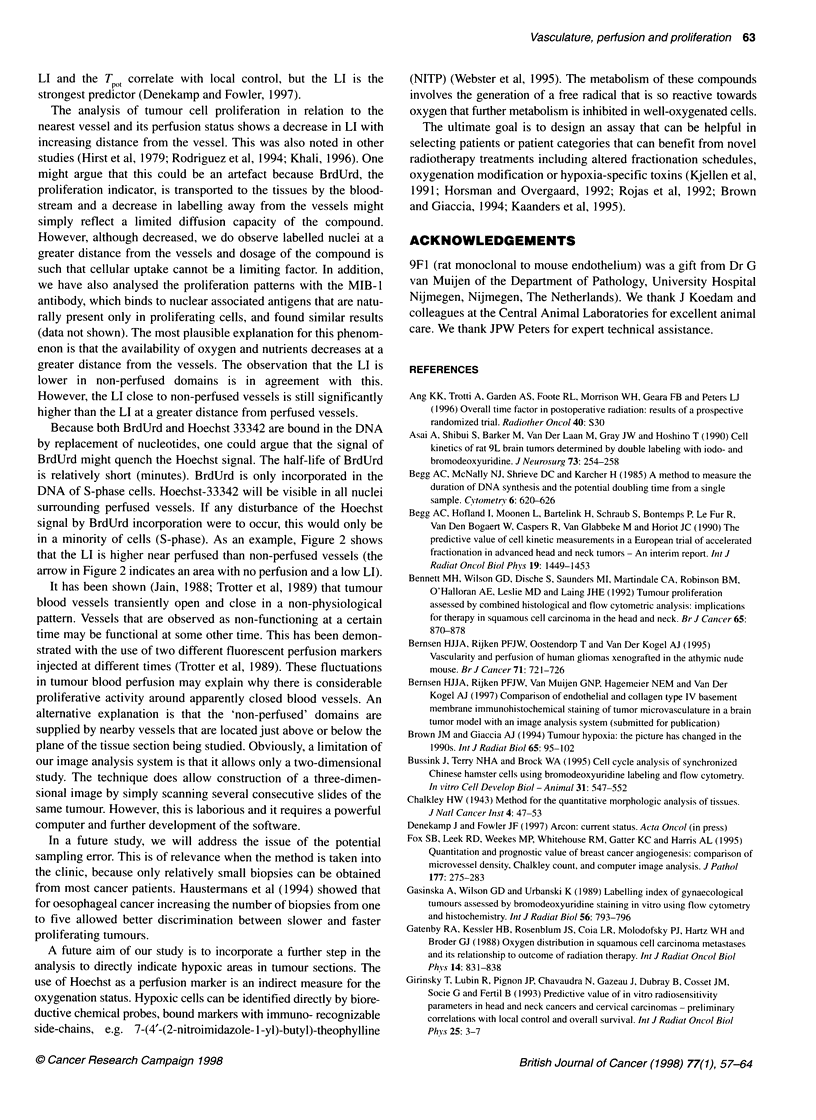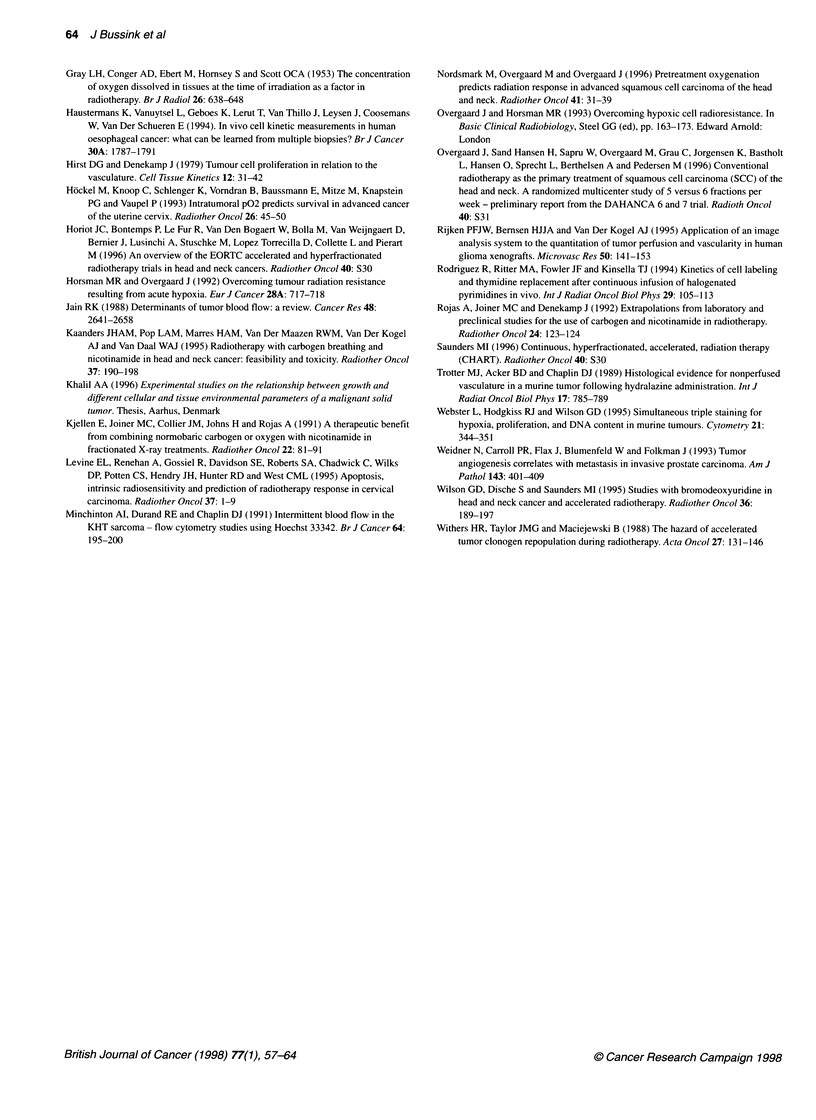# Multiparameter analysis of vasculature, perfusion and proliferation in human tumour xenografts.

**DOI:** 10.1038/bjc.1998.9

**Published:** 1998

**Authors:** J. Bussink, J. H. Kaanders, P. F. Rijken, C. A. Martindale, A. J. van der Kogel

**Affiliations:** Institute of Radiotherapy, University of Nijmegen, The Netherlands.

## Abstract

**Images:**


British Journal of Cancer (1998) 77(1), 57–64
© 1998 Cancer Research Campaign

### Multiparameter analysis of vasculature, perfusion and proliferation in human tumour xenografts

**J Bussink[1], JHAM Kaanders[1], PFJW Rijken[1], CA Martindale[2] and AJ van der Kogel[1]**

[1]Institute of Radiotherapy, University of Nijmegen, Geert Grooteplein 32, PO Box 9101, 6500 HB Nijmegen, The Netherlands; [2]Gray Laboratory Cancer Research Trust, PO Box 100, Mount Vernon Hospital, Northwood, Middlesex, HA6 2JR, UK

**Summary** A method is presented in this report for concurrent analysis of vascular architecture, blood perfusion and proliferation characteristics in whole-tumour cross-sections of human larynx carcinoma and glioblastoma xenografts. Tumours were implanted subcutaneously in nude mice. After i.v. injection with Hoechst 33342 and bromodeoxyuridine (BrdUrd) as perfusion and proliferation markers, animals were killed. An antiendothelial antibody (9F1) was used to delineate vascular structures. Cross-sections were analysed by a multistep immune staining and a computer-controlled microscope scanning method. Each tumour section was stained and scanned four times (Hoechst, 9F1, BrdUrd and Fast Blue for all nuclei). When these images were combined, vasculature, perfusion and proliferation parameters were analysed. The labelling index (LI) was defined as the ratio of the BrdUrd-labelled area to the total nuclear area. The LI based on manual counting and the LI calculated by flow cytometry (FCM) were in good agreement with the LI based on surface analysis. LI decreased at increasing distance from its nearest vessel. In the vicinity of perfused vessels, the LI was 30–70% higher than near non-perfused vessels. This method shows that both vasculature/perfusion and proliferation characteristics can be measured in the same whole-tumour section in a semiautomatic way. This could be applied in clinical practice to identify combined human tumour characteristics that predict for a favourable response to treatment modifications.

**Keywords:** squamous cell carcinoma; glioblastoma; image analysis; proliferation; vasculature; perfusion

The radiation response of tumours is determined by several well-recognized factors, including intrinsic radio sensitivity, cell kinetics and the degree of tumour oxygenation and perfusion. There is increasing recognition that a combination of these mechanisms may be responsible for treatment failure in certain tumour types.

The prognostic relevance of intrinsic radio sensitivity, measured by clonogenic assays, has been demonstrated for cancer of the uterine cervix (Levine et al, 1995) and for head and neck cancer (Girinsky et al, 1993).

Another cause for radiation treatment failure is tumour cell repopulation, which compensates for radiation-induced cell kill. The longer the overall treatment time of fractionated radiation treatments, the greater the opportunity for tumour cell repopulation. Withers et al (1988) have reviewed 59 clinical studies on head and neck cancer and have demonstrated that the outcome was worse with longer treatment schedules. Preliminary results from four randomized studies in head and neck cancer and one in bronchus carcinoma demonstrate that tumour control rates can be improved with shortened radiation treatment schedules delivering two or more radiation fractions per day relative to once-a-day treatments with conventional radiotherapy (Ang et al, 1996; Horiot et al, 1996; Overgaard et al, 1996; Saunders, 1996).

Begg et al (1990) measured the potential doubling time ($T_{pot}$) in biopsies of head and neck carcinomas by flow cytometry (FCM) and showed that this could be predictive for treatment outcome. A trend was shown for the fast proliferating tumours to perform worse on a conventional treatment schedule than slow proliferating tumours. The outcome of the fast proliferating tumours was improved with accelerated radiotherapy, although the differences were not statistically significant. In data collected from ten different European trials, Begg et al (1990) showed that the pretreatment kinetic parameters, analysed by flow cytometry, can predict local control after conventional radiotherapy (Denekamp and Fowler, 1997). Bennett et al (1992) categorized squamous cell carcinomas by histological proliferation patterns that appeared to be correlated with radiation treatment outcome.

A third factor determining the effect of radiotherapy is oxygenation. Radiotherapy is less effective under hypoxic conditions (Gray et al, 1953; Minchinton et al, 1991). Hypoxia exists in varying degrees in nearly all tumours. In advanced cancer of the uterine cervix and in nodal metastases of head and neck tumours, intratumoral $pO_2$, as detected by polarographic measurements, predicted for survival (Gatenby et al, 1988; Höckel et al, 1993; Nordsmark et al, 1996).

A variety of procedures have been developed to overcome hypoxic radiation resistance including high oxygen content gas breathing under normo- or hyperbaric conditions, hypoxic cell radiosensitizers, blood transfusions and use of vasoactive drugs. A meta-analysis of 83 randomized clinical trials showed that both local control and survival can be improved by reduction of hypoxia (Overgaard and Horsman, 1993).

Tumour hypoxia is the result of a chaotically organized vascular network and an insufficient and heterogeneous blood supply. A detailed understanding of the vascular architecture and perfusion status of tumours is necessary to develop and assess methods for tumour oxygenation modification in an effort to improve radiation

*Received 28 February 1997*
*Revised 9 June 1997*
*Accepted 12 June 1997*

*Correspondence to:* J Bussink response. In addition, it has been shown that the metastatic potential of tumours is related to certain vascular parameters (Weidner et al, 1993). The morphological aspects are commonly analysed using stereological principles such as the point-counting method (Chalkley et al, 1943; Fox et al, 1995). Information on vascular function is obtained using radioactive and fluorescent perfusion markers (Jain, 1988).

Bernsen et al (1995) presented a method to analyse quantitatively both morphological parameters of vascular architecture and perfusion using an automated image analysis system. This paper presents a further development of this method that includes the simultaneous measurement of kinetic parameters.

The aim of this study was to standardize a method for quantitative analysis of vasculature, blood perfusion and proliferation in the same tissue section. This could be applied in clinical practice to identify the combined human tumour characteristics that predict for a favourable response to treatment modifications, including oxygen modification, altered fractionation schedules and combinations of these approaches.

### MATERIALS AND METHODS

### Tumours

Tumours were derived from different primary human high-grade gliomas and head and neck squamous cell carcinomas (moderately to well-differentiated). Viable 1-mm$^3$ tumour pieces were transplanted subcutaneously in nude mice (Balb/c nu/nu mouse). Tumours were passaged when they reached a diameter of 1–1.5 cm. For the analysis of vascular and kinetic parameters, tumours with a diameter of 0.6–1.0 cm were used.

### Markers of proliferation and perfusion

The S-phase marker bromodeoxyuridine (BrdUrd) (Sigma Chemical, St Louis, MO, USA) was given at a dose of 100 mg kg$^1$ intraperitoneally, 15 min before the animals were killed. In experiments in which the in situ analysis is compared with flow cytometry (FCM), BrdUrd was injected 5 h before killing the animals to allow calculation of the potential doubling time ($T_{pot}$) by the relative movement method (Begg et al, 1985). Two minutes before killing the animals, Hoechst 33342 was given intravenously via one of the tail veins as a marker of perfusion. Tumour specimens were cut in two: half was stored in liquid nitrogen until frozen sections were cut, which were then stored at –80°C until staining and the other half was fixed in 70% ethanol for FCM analysis.

### Immunohistochemical staining

After thawing, sections of 5 μm thickness were fixed in acetone for 10 min and slides were then rinsed and mounted in phosphate-buffered saline (PBS). Then, the tissue sections were scanned for the Hoechst signal. The starting point and the resulting composite binary image with perfused tissue areas were both stored in the computer. Details on the scanning procedure are given below. The same sections were then stained for endothelial structures. First, they were incubated for 45 min at room temperature with undiluted 9F1 (rat monoclonal antibody to mouse endothelium, Department of Pathology, University Hospital Nijmegen, The Netherlands). After rinsing, the sections were then incubated for 30 min at room temperature with tetramethylrhodamine isothiocyanate (TRITC)-conjugated rabbit anti-rat antibodies (Organon Teknick, West Chester, PA, USA) diluted 1:100 in phosphate-buffered saline (PBS) with 10% normal mouse serum followed by rinsing and 30 min incubation at room temperature with TRITC-conjugated goat anti-rabbit (Tago, Burlingame, CA, USA) diluted 1:50 in PBS with 1% BSA. The sections were then rinsed again.

Next, BrdUrd was immunohistochemically visualized. The DNA of the tissue sections was denatured by incubation with 2 N hydrochloric acid for 10 min. To neutralize the pH, sections were rinsed in 0.1 M borax for 10 min followed by rinsing in PBS. The sections were then incubated for 60 min at 37 °C with Br-3 (mouse monoclonal to BrdUrd, Caltag Laboratories S. San Francisco, CA, USA) diluted 1:50 in PBT (PBS with 1% BSA and 0.5% Tween-20). Next, after rinsing with PBS again, the sections were incubated for 45 min at room temperature with fluorescein isothiocyanate (FITC)-conjugated rat anti-mouse antibodies (Dako, Denmark) diluted 1:25 in PBT.

As a final step, all nuclei were stained with Fast Blue (Sigma) diluted 1:1000 in PBS for 15 min at room temperature and finally rinsed again.

### Scanning of tumour sections and image processing

The tumour sections were scanned by a computer-controlled procedure using a high-resolution intensified solid-state camera for quantitative analysis on a Zeiss microscope. A detailed description of this scanning method has been given by Rijken et al (1995). Each tumour section was sequentially scanned four times, at 200 × magnification, using different filters (TRITC signal, 510–560 nm excitation and 690 nm emission filter; FITC, 450–490 nm excitation and 520 nm emission filter; Hoechst and Fast Blue, 365 nm excitation and 420 nm emission filter). After processing all fields (scanning in eight times eight steps, i.e. 64 fields with a field size of 0.31 mm$^2$), the scanned area was reconstructed from the separate processed images into one large composite image. The result of this scanning procedure is four composite images: one showing the perfused areas (Hoechst), one showing vascular structures (9F1), one showing proliferating nuclei (BrdUrd) and one showing all nuclei (Fast Blue). As a final step, the tumour area was determined by drawing a contour line. This area was used as a mask in further image analysis, excluding non-tumour tissue and necrotic areas from the analysis. Stromal cells that are close to tumour cells or embedded in the tumour tissue (for instance near vascular structures) cannot be excluded from the analysis.

### Analysis of vascular and kinetic parameters

The vascular parameters can be derived from the first two composite images (Hoechst and 9F1) (Bernsen et al, 1995). When these two images are combined, the overlapping structures represent those vascular structures that were perfused at the time of injection of Hoechst; the vascular structures that have no corresponding Hoechst staining of adjacent nuclei represent non-perfused vessels. The area of perfused vascular structures divided by the total vascular area of the tissue section yields the perfusion fraction, indicating the fraction of vascular structures that was perfused at the time of Hoechst injection.

Because of the high cellular density of most tumours in the tissue sections, nuclei are often abutting or overlapping and individual

 © Cancer Research Campaign 1998

**Figure 1** The principle of vascular domains: the tissue section is divided into areas on the basis of vascular structure, domains. The boundaries of these domains are equidistant to adjacent vessels

**Figure 3** LI after computerized in situ analysis vs FCM; the samples of the aneuploid tumours are indicated by asterisks nuclei cannot always be discriminated by the scanning system. It is therefore not possible to calculate the labelling index (LI), which is the number of BrdUrd-positive nuclei relative to the total number of nuclei, based on the count of individual labelled and unlabelled nuclei. Therefore, the LI was determined from the ratio of the BrdUrd-positive surface (FITC) to the total nuclear surface (Fast Blue). To validate this method, the same microscopic fields were analysed by the computer-controlled analysis system and by manual counting, both at 400 × magnification. With the manual count, at least 200 nuclei were counted in 15 microscope fields of a glioblastoma tumour line. The LI obtained by the two methods were compared.

The heterogeneity of distribution of proliferating cells within a tumour section was assessed by dividing the composite image in 16 × 16 fields, thus yielding 256 arbitrary tissue areas of 0.076 $mm^2$ each, corresponding to approximately one high-power microscope field of 400 × magnification. For each of these individual areas the LI was calculated and the mean LI of the 256 tissue areas with standard deviation were obtained for each tissue section. In addition, the overall LI for the whole section was calculated.

For a better understanding of the relationship between proliferation and vascularity, the composite images were subdivided into functional units, so-called 'vascular domains'. A domain was constructed around each vascular structure in a separate image. The boundaries of these domains were defined such that they were

**Figure 2** The process of staining and scanning of the same tissue section (squamous cell carcinoma tumour line PC3). Binary reconstructed image after scanning a tissue section four times. Hoechst for perfusion (blue), 9F1 TRITC for vascular structures (red), BrdUrd FITC for proliferation (green). The Fast Blue signal for the total nuclear surface is not shown. Note non-perfused vascular structures (solid arrow) and perfused vascular structures and the decrease in LI at increasing distance from vessels equidistant to adjacent vessels (Figure 1). In the recorded composite images, the LI was calculated in four arbitrary zones at increasing distance from the surface of the vessel in each domain: 0–15 μm, 15–30 μm, 30–70 μm and > 70 μm from the nearest vessel. The LI was analysed for every individual domain, distinguishing perfused and non-perfused domains.

### Flow cytometry

The method of staining and analysis using flow cytometry (FCM) has been described elsewhere (Bennett et al, 1992). Briefly, ethanol-fixed tissue fragments were digested into nuclei using 0.4 mg $ml^{-1}$ pepsin in 0.1 M hydrochloric acid for 30 min at 37 °C. DNA was denatured with 2 M hydrochloric acid for 12 min at room temperature. The nuclei were incubated with Br-3 FITC conjugate (mouse monoclonal to BrdUrd, Caltag Laboratories) in PBS containing 0.5% Tween-20 and 0.5% normal goat serum for 2 h at room temperature. Total DNA was stained using 10 μg $ml^{-1}$ propidium iodide and the samples were analysed by FCM. Samples were run on a Becton Dickinson FACScan with a single excitation wavelength of 488 nm. Doublets were excluded by gating on the width and area signals from the FL3 channel. Ten thousand events were collected. The data derived from the FCM profiles were the DNA index, the LI of all cells and of the aneuploid subcompartment in appropriate tumours making a correction for cell division (Wilson et al, 1995). The DNA synthesis time ($T_S$) was calculated using the method of Begg et al (1985) and the $T_{pot}$ was derived using the formula: $T_{pot} = \lambda \times T_S/LI$, with $\lambda = 0.8$.

### Statistics

The LI obtained by the scanning method based on labelled surface analysis was compared with the LI obtained by manual counting and by FCM using linear least-squares regression analysis. The *t*-test was used to compare LI in perfused domains with the LI in non-perfused domains. For analysis of the decrease in LI at increasing distance from vascular structures, MANCOVA was used for all data points. To determine where these differences in the means of any two distance intervals were present, the post-hoc Tukey honest significant difference (HSD) test for multiple comparisons was applied.

**Figure 4** LI after computerized in situ analysis vs manual count

### RESULTS

### Images

Figure 2 shows the composite binary image obtained after the scanning procedure for a representative tumour section. The combination of the Hoechst, 9F1 and BrdUrd images clearly shows the distribution of proliferating cells throughout the tissue section and the relation to vascular structures (Figure 2). Note perfused and non-perfused vascular structures and the decrease in LI at increasing distance from vessels.

**Table 1** FCM analysis vs computerized in situ analysis

| Tumour | Flow cytometry | | | Computerized in situ analysis | | | |
|---|---|---|---|---|---|---|---|
| | $T_{pot}$ (days)[a] | LI all cells | LI aneuploid cells | LI in situ[b] | Mean[c] | s.d.[c] | Range[c] |
| 1 GM2 | 2.6 | 0.13 | | 0.110 | 0.11 | 0.072 | 0–0.32 |
| 2 GM2 | – | – | | 0.059 | 0.033 | 0.032 | 0–0.16 |
| 3 GM34 | 7.0 | 0.041 | 0.064 | 0.05 | 0.040 | 0.034 | 0–0.17 |
| 4 GM34 | – | – | | 0.024 | 0.023 | 0.026 | 0–0.11 |
| 5 GM49 | 3.3 | 0.088 | 0.094 | 0.055 | 0.054 | 0.037 | 0–0.17 |
| 6 GM106 | 3.7 | 0.080 | 0.086 | 0.10 | 0.10 | 0.11 | 0–0.40 |
| 7 GM106 | 2.0 | 0.12 | 0.15 | 0.16 | 0.18 | 0.080 | 0–0.36 |
| 8 GM182 | 2.6 | 0.092 | 0.11 | – | – | – | – |
| 9 GM192 | 8.9 | 0.034 | | 0.038 | 0.027 | 0.029 | 0–0.14 |
| 10 PC3p3 | – | – | | 0.043 | 0.015 | 0.013 | 0–0.39 |
| 11 PC3p3 | – | – | | 0.016 | 0.013 | 0.015 | 0–0.059 |
| 12 PC20 | 5.2 | 0.046 | 0.054 | 0.050 | 0.032 | 0.031 | 0–0.12 |
| 13 PC18 | – | – | | – | – | – | – |
| 14 PC3p4 | 1.8 | 0.095 | | – | – | – | – |
| 15 PC3p4 | 8.3 | 0.033 | | 0.063 | 0.060 | 0.049 | 0–0.21 |
| 16 PC3p4 | 5.6 | 0.056 | | 0.075 | 0.073 | 0.0059 | 0–0.26 |
| 17 PC3p4 | 3.1 | 0.075 | | – | – | – | – |
| 18 PC3p4 | – | – | | 0.037 | 0.023 | 0.027 | 0–0.094 |

GM, glioblastoma multiforme; PC, squamous cell carcinoma. [a]$T_{pot}$ is calculated on the basis of the aneuploid cells if present, otherwise on the diploid cell population. [b]Overall LI of a whole tissue section. [c]Mean LI, standard deviation (SD) and range of 256 tissue areas of one tissue section.

*© Cancer Research Campaign 1998*

**Figure 5** Example of the distribution of LI in 256 fields each representing 0.076 mm² of a glioblastoma tissue section

### BrdUrd LI: comparing three methods

Calculation of LI based on computerized in situ analysis as the ratio of BrdUrd-labelled nuclear surface to the total nuclear surface was compared with manual counting and with FCM results.

For 12 tumours from seven different tumour lines, the LI obtained by FCM was compared with the computerized in situ method. From three samples it was not possible to obtain a reliable result by computerized in situ analysis because of heavy background staining that made it impossible to analyse the tumour section. One tumour consisted almost completely of necrosis and could not be analysed by either computerized in situ analysis or FCM. In nine out of these 12 tumours both in situ and FCM results were available, showing a good correlation between the two methods (correlation coefficient 0.81, Figure 3). Only six of these tumours were aneuploid: of these six, the correlation with the computerized in situ analysis showed a correlation coefficient of 0.90. Analysis by FCM also yielded $T_{pot}$ values, which are given in Table 1.

A good correlation was observed between the computerized surface-based LI and the LI based on manual count (correlation coefficient 0.89, $P < 0.05$, Figure 4).

### Distribution of LI and relation to vasculature

Both the inter- and intratumour variability of LI are large (Table 1). As an example of intratumour variability, the distribution profile of the LI in a high-grade glioma is given in Figure 5 (number 1 in Table 1).

After construction of the vascular domains, the LI was calculated at different distances from the surface of the nearest vessel. Figure 6 shows the results obtained in three different squamous cell carcinoma tumour lines. Each panel represents the average LI of 4–5 tumours of the same tumour line. At increasing distance from the nearest vessel, the LI decreases both in perfused and non-perfused vascular domains. The LI calculated for the cells that are nearest to the vessel is based on the area that also includes the extravascular matrix. In this matrix, slower proliferating cells, such as fibroblasts, are present, and this is the reason for the lower LI in the 0–15 μm range. There is a statistically significant decrease in LI at increasing distance in all three tumour lines with $P$-values < 0.05 if the LI from 15–30 μm is compared with the interval > 70 μm (Tukey HSD test).

Overall, the LI is lower in non-perfused domains than in perfused domains (Figure 7).

**Figure 6** LI vs distance from the nearest vessel, average of five tumours with SEM. PC3, PC15 and PC19 are different human laryngeal squamous cell carcinoma tumour lines

### DISCUSSION

This paper describes how the semiautomatic method for quantitative analysis of tumour perfusion and vasculature, as described by Rijken et al (1995), was further developed to allow also quantitation of proliferative activity in the same tumour section. Without disturbing tissue architecture, the proliferative patterns of tumours can be studied and related to vascularity with the possibility of distinguishing between functional and non-functional vessels.

For detection of vascular structures the newly developed anti-mouse endothelium antibody, 9F1, was used. It recognizes the

© Cancer Research Campaign 1998

**Figure 7** Average LI of four (PC19) or five (PC3 and PC15) tumours. Perfused domains (■) and non-perfused domains (▨) at four different zones around vessels. **A**, 0–15 μm; **B**, 15–30 μm; **C**, 30–70 μm; and **D**, > 70 μm. Error bars indicate s.e.m. (**t*-test, $P < 0.05$).

same vascular structures as the more commonly used collagen IV antibody (HJJA Bernsen et al, submitted for publication). In the squamous cell carcinoma xenografts, however, the collagen IV antibody produces significant background staining because of considerable amounts of collagen in the extracellular compartment of these tumours. This background staining is less with the 9F1 antibody, which is therefore more suitable for the automated image analysis.

The LI based on computerized surface analysis is in good agreement with the LI based on manual counting and FCM analysis. Other studies have also found a good correlation between LI based on FCM and manual counting (Gasinska et al, 1989; Bennett et al, 1992; Bussink et al, 1995).

FCM has the advantage that large cell numbers can be counted. However, this method produces an average LI for the entire tissue sample and information on spatial heterogeneity of proliferative activity within a tumour is lost. In addition, in diploid tumours FCM cannot distinguish tumour cells from normal cells, which may significantly influence the analysis. Assessment of LI by computerized surface analysis gives a lower LI than manual counting of labelled nuclei (difference approximately 0.05, Figure 3). This difference was also seen when the LI of tumour sections was compared with the LI obtained by FCM (Bennett et al, 1992).

This difference could be caused by non-tumour cells in the tumour section. Assessment of the LI by computerized surface analysis can be influenced by the thresholding of the FITC signal. If the threshold is set too high, BrdUrd-positive cells are not identified and will result in a lower LI. This can also introduce a systemic difference between FCM measurements and our method.

Another advantage of the immunohistochemical method over FCM is that it allows the study of tumour cell kinetics in relation to histology. It has been suggested that the histological proliferation pattern can be a stronger predictor for clinical outcome than the LI or $T_{pot}$ (Bennett et al, 1992; Wilson et al, 1995). Manual counting in tissue sections is time-consuming, however, and only limited cell numbers can be analysed. In addition, there is an element of subjectivity with interobserver variations. The computer-controlled scanning method combines the strengths of FCM and manual counting: proliferation patterns can be analysed rapidly in complete tissue sections. In addition, the translation into binary images greatly facilitates quantitation of any of the parameters being studied (proliferation, vascular patterns and perfusion). Two-dimensional analysis allows spatial study of relationships between (functional) vessels and cell proliferation. Areas of interest can be chosen for detailed analysis of non-tumour tissue and necrotic areas can be easily excluded. FCM allows calculation of $T_{pot}$ because it also gives information on the DNA content of nuclei (Begg et al, 1985). Calculation of $T_{pot}$ with the immunohistochemical method would require two consecutive injections with thymidine analogues, e.g. BrdUrd and IdUrd, to allow estimation of S-phase duration (Asai et al, 1990). We have attempted this, but with the computer analysis system double-labelled nuclei are not always reliably distinguished from single-labelled nuclei and technical improvements are required to enable estimation of $T_{pot}$ by this method. In contrast, there is currently some debate as to whether $T_{pot}$ has any additional value over LI as a predictor of radiotherapy outcome. In data collected from ten different European trials, Begg et al (1990) showed that both the

 © Cancer Research Campaign 1998

LI and the $T_{pot}$ correlate with local control, but the LI is the strongest predictor (Denekamp and Fowler, 1997).

The analysis of tumour cell proliferation in relation to the nearest vessel and its perfusion status shows a decrease in LI with increasing distance from the vessel. This was also noted in other studies (Hirst et al, 1979; Rodriguez et al, 1994; Khali, 1996). One might argue that this could be an artefact because BrdUrd, the proliferation indicator, is transported to the tissues by the bloodstream and a decrease in labelling away from the vessels might simply reflect a limited diffusion capacity of the compound. However, although decreased, we do observe labelled nuclei at a greater distance from the vessels and dosage of the compound is such that cellular uptake cannot be a limiting factor. In addition, we have also analysed the proliferation patterns with the MIB-1 antibody, which binds to nuclear associated antigens that are naturally present only in proliferating cells, and found similar results (data not shown). The most plausible explanation for this phenomenon is that the availability of oxygen and nutrients decreases at a greater distance from the vessels. The observation that the LI is lower in non-perfused domains is in agreement with this. However, the LI close to non-perfused vessels is still significantly higher than the LI at a greater distance from perfused vessels.

Because both BrdUrd and Hoechst 33342 are bound in the DNA by replacement of nucleotides, one could argue that the signal of BrdUrd might quench the Hoechst signal. The half-life of BrdUrd is relatively short (minutes). BrdUrd is only incorporated in the DNA of S-phase cells. Hoechst-33342 will be visible in all nuclei surrounding perfused vessels. If any disturbance of the Hoechst signal by BrdUrd incorporation were to occur, this would only be in a minority of cells (S-phase). As an example, Figure 2 shows that the LI is higher near perfused than non-perfused vessels (the arrow in Figure 2 indicates an area with no perfusion and a low LI).

It has been shown (Jain, 1988; Trotter et al, 1989) that tumour blood vessels transiently open and close in a non-physiological pattern. Vessels that are observed as non-functioning at a certain time may be functional at some other time. This has been demonstrated with the use of two different fluorescent perfusion markers injected at different times (Trotter et al, 1989). These fluctuations in tumour blood perfusion may explain why there is considerable proliferative activity around apparently closed blood vessels. An alternative explanation is that the 'non-perfused' domains are supplied by nearby vessels that are located just above or below the plane of the tissue section being studied. Obviously, a limitation of our image analysis system is that it allows only a two-dimensional study. The technique does allow construction of a three-dimensional image by simply scanning several consecutive slides of the same tumour. However, this is laborious and it requires a powerful computer and further development of the software.

In a future study, we will address the issue of the potential sampling error. This is of relevance when the method is taken into the clinic, because only relatively small biopsies can be obtained from most cancer patients. Haustermans et al (1994) showed that for oesophageal cancer increasing the number of biopsies from one to five allowed better discrimination between slower and faster proliferating tumours.

A future aim of our study is to incorporate a further step in the analysis to directly indicate hypoxic areas in tumour sections. The use of Hoechst as a perfusion marker is an indirect measure for the oxygenation status. Hypoxic cells can be identified directly by bioreductive chemical probes, bound markers with immuno- recognizable side-chains, e.g. 7-(4′-(2-nitroimidazole-1-yl)-butyl)-theophylline (NITP) (Webster et al, 1995). The metabolism of these compounds involves the generation of a free radical that is so reactive towards oxygen that further metabolism is inhibited in well-oxygenated cells.

The ultimate goal is to design an assay that can be helpful in selecting patients or patient categories that can benefit from novel radiotherapy treatments including altered fractionation schedules, oxygenation modification or hypoxia-specific toxins (Kjellen et al, 1991; Horsman and Overgaard, 1992; Rojas et al, 1992; Brown and Giaccia, 1994; Kaanders et al, 1995).

### ACKNOWLEDGEMENTS

9F1 (rat monoclonal to mouse endothelium) was a gift from Dr G van Muijen of the Department of Pathology, University Hospital Nijmegen, Nijmegen, The Netherlands). We thank J Koedam and colleagues at the Central Animal Laboratories for excellent animal care. We thank JPW Peters for expert technical assistance.

### REFERENCES

Ang KK, Trotti A, Garden AS, Foote RL, Morrison WH, Geara FB and Peters LJ (1996) Overall time factor in postoperative radiation: results of a prospective randomized trial. *Radiother Oncol* **40**: S30

Asai A, Shibui S, Barker M, Van Der Laan M, Gray JW and Hoshino T (1990) Cell kinetics of rat 9L brain tumors determined by double labeling with iodo- and bromodeoxyuridine. *J Neurosurg* **73**: 254–258

Begg AC, McNally NJ, Shrieve DC and Karcher H (1985) A method to measure the duration of DNA synthesis and the potential doubling time from a single sample. *Cytometry* **6**: 620–626

Begg AC, Hofland I, Moonen L, Bartelink H, Schraub S, Bontemps P, Le Fur R, Van Den Bogaert W, Caspers R, Van Glabbeke M and Horiot JC (1990) The predictive value of cell kinetic measurements in a European trial of accelerated fractionation in advanced head and neck tumors – An interim report. *Int J Radiat Oncol Biol Phys* **19**: 1449–1453

Bennett MH, Wilson GD, Dische S, Saunders MI, Martindale CA, Robinson BM, O'Halloran AE, Leslie MD and Laing JHE (1992) Tumour proliferation assessed by combined histological and flow cytometric analysis: implications for therapy in squamous cell carcinoma in the head and neck. *Br J Cancer* **65**: 870–878

Bernsen HJJA, Rijken PFJW, Oostendorp T and Van Der Kogel AJ (1995) Vascularity and perfusion of human gliomas xenografted in the athymic nude mouse. *Br J Cancer* **71**: 721–726

Bernsen HJJA, Rijken PFJW, Van Muijen GNP, Hagemeier NEM and Van Der Kogel AJ (1997) Comparison of endothelial and collagen type IV basement membrane immunohistochemical staining of tumor microvasculature in a brain tumor model with an image analysis system (submitted for publication)

Brown JM and Giaccia AJ (1994) Tumour hypoxia: the picture has changed in the 1990s. *Int J Radiat Biol* **65**: 95–102

Bussink J, Terry NHA and Brock WA (1995) Cell cycle analysis of synchronized Chinese hamster cells using bromodeoxyuridine labeling and flow cytometry. *In vitro Cell Develop Biol – Animal* **31**: 547–552

Chalkley HW (1943) Method for the quantitative morphologic analysis of tissues. *J Natl Cancer Inst* **4**: 47–53

Denekamp J and Fowler JF (1997) Arcon: current status. *Acta Oncol* (in press)

Fox SB, Leek RD, Weekes MP, Whitehouse RM, Gatter KC and Harris AL (1995) Quantitation and prognostic value of breast cancer angiogenesis: comparison of microvessel density, Chalkley count, and computer image analysis. *J Pathol* **177**: 275–283

Gasinska A, Wilson GD and Urbanski K (1989) Labelling index of gynaecological tumours assessed by bromodeoxyuridine staining in vitro using flow cytometry and histochemistry. *Int J Radiat Biol* **56**: 793–796

Gatenby RA, Kessler HB, Rosenblum JS, Coia LR, Molodofsky PJ, Hartz WH and Broder GJ (1988) Oxygen distribution in squamous cell carcinoma metastases and its relationship to outcome of radiation therapy. *Int J Radiat Oncol Biol Phys* **14**: 831–838

Girinsky T, Lubin R, Pignon JP, Chavaudra N, Gazeau J, Dubray B, Cosset JM, Socie G and Fertil B (1993) Predictive value of in vitro radiosensitivity parameters in head and neck cancers and cervical carcinomas – preliminary correlations with local control and overall survival. *Int J Radiat Oncol Biol Phys* **25**: 3–7

© Cancer Research Campaign 1998

Gray LH, Conger AD, Ebert M, Hornsey S and Scott OCA (1953) The concentration of oxygen dissolved in tissues at the time of irradiation as a factor in radiotherapy. *Br J Radiol* **26**: 638–648

Haustermans K, Vanuytsel L, Geboes K, Lerut T, Van Thillo J, Leysen J, Coosemans W, Van Der Schueren E (1994). In vivo cell kinetic measurements in human oesophageal cancer: what can be learned from multiple biopsies? *Br J Cancer* **30A**: 1787–1791

Hirst DG and Denekamp J (1979) Tumour cell proliferation in relation to the vasculature. *Cell Tissue Kinetics* **12**: 31–42

Höckel M, Knoop C, Schlenger K, Vorndran B, Baussmann E, Mitze M, Knapstein PG and Vaupel P (1993) Intratumoral pO2 predicts survival in advanced cancer of the uterine cervix. *Radiother Oncol* **26**: 45–50

Horiot JC, Bontemps P, Le Fur R, Van Den Bogaert W, Bolla M, Van Weijngaert D, Bernier J, Lusinchi A, Stuschke M, Lopez Torrecilla D, Collette L and Pierart M (1996) An overview of the EORTC accelerated and hyperfractionated radiotherapy trials in head and neck cancers. *Radiother Oncol* **40**: S30

Horsman MR and Overgaard J (1992) Overcoming tumour radiation resistance resulting from acute hypoxia. *Eur J Cancer* **28A**: 717–718

Jain RK (1988) Determinants of tumor blood flow: a review. *Cancer Res* **48**: 2641–2658

Kaanders JHAM, Pop LAM, Marres HAM, Van Der Maazen RWM, Van Der Kogel AJ and Van Daal WAJ (1995) Radiotherapy with carbogen breathing and nicotinamide in head and neck cancer: feasibility and toxicity. *Radiother Oncol* **37**: 190–198

Khalil AA (1996) *Experimental studies on the relationship between growth and different cellular and tissue environmental parameters of a malignant solid tumor*. Thesis, Aarhus, Denmark

Kjellen E, Joiner MC, Collier JM, Johns H and Rojas A (1991) A therapeutic benefit from combining normobaric carbogen or oxygen with nicotinamide in fractionated X-ray treatments. *Radiother Oncol* **22**: 81–91

Levine EL, Renehan A, Gossiel R, Davidson SE, Roberts SA, Chadwick C, Wilks DP, Potten CS, Hendry JH, Hunter RD and West CML (1995) Apoptosis, intrinsic radiosensitivity and prediction of radiotherapy response in cervical carcinoma. *Radiother Oncol* **37**: 1–9

Minchinton AI, Durand RE and Chaplin DJ (1991) Intermittent blood flow in the KHT sarcoma – flow cytometry studies using Hoechst 33342. *Br J Cancer* **64**: 195–200

Nordsmark M, Overgaard M and Overgaard J (1996) Pretreatment oxygenation predicts radiation response in advanced squamous cell carcinoma of the head and neck. *Radiother Oncol* **41**: 31–39

Overgaard J and Horsman MR (1993) Overcoming hypoxic cell radioresistance. In *Basic Clinical Radiobiology*, Steel GG (ed), pp. 163–173. Edward Arnold: London

Overgaard J, Sand Hansen H, Sapru W, Overgaard M, Grau C, Jorgensen K, Bastholt L, Hansen O, Sprecht L, Berthelsen A and Pedersen M (1996) Conventional radiotherapy as the primary treatment of squamous cell carcinoma (SCC) of the head and neck. A randomized multicenter study of 5 versus 6 fractions per week – preliminary report from the DAHANCA 6 and 7 trial. *Radioth Oncol* **40**: S31

Rijken PFJW, Bernsen HJJA and Van Der Kogel AJ (1995) Application of an image analysis system to the quantitation of tumor perfusion and vascularity in human glioma xenografts. *Microvasc Res* **50**: 141–153

Rodriguez R, Ritter MA, Fowler JF and Kinsella TJ (1994) Kinetics of cell labeling and thymidine replacement after continuous infusion of halogenated pyrimidines in vivo. *Int J Radiat Oncol Biol Phys* **29**: 105–113

Rojas A, Joiner MC and Denekamp J (1992) Extrapolations from laboratory and preclinical studies for the use of carbogen and nicotinamide in radiotherapy. *Radiother Oncol* **24**: 123–124

Saunders MI (1996) Continuous, hyperfractionated, accelerated, radiation therapy (CHART). *Radiother Oncol* **40**: S30

Trotter MJ, Acker BD and Chaplin DJ (1989) Histological evidence for nonperfused vasculature in a murine tumor following hydralazine administration. *Int J Radiat Oncol Biol Phys* **17**: 785–789

Webster L, Hodgkiss RJ and Wilson GD (1995) Simultaneous triple staining for hypoxia, proliferation, and DNA content in murine tumours. *Cytometry* **21**: 344–351

Weidner N, Carroll PR, Flax J, Blumenfeld W and Folkman J (1993) Tumor angiogenesis correlates with metastasis in invasive prostate carcinoma. *Am J Pathol* **143**: 401–409

Wilson GD, Dische S and Saunders MI (1995) Studies with bromodeoxyuridine in head and neck cancer and accelerated radiotherapy. *Radiother Oncol* **36**: 189–197

Withers HR, Taylor JMG and Maciejewski B (1988) The hazard of accelerated tumor clonogen repopulation during radiotherapy. *Acta Oncol* **27**: 131–146

© Cancer Research Campaign 1998